# Symptom-severity-related brain connectivity alterations in functional movement disorders

**DOI:** 10.1016/j.nicl.2022.102981

**Published:** 2022-03-03

**Authors:** Karsten Mueller, Filip Růžička, Matěj Slovák, Zuzana Forejtová, Petr Dušek, Pavel Dušek, Robert Jech, Tereza Serranová

**Affiliations:** a Max Planck Institute for Human Cognitive and Brain Sciences, Leipzig, Germany; b Department of Neurology and Center of Clinical Neuroscience, Charles University in Prague, First Faculty of Medicine and General University Hospital in Prague, Czech Republic

**Keywords:** Functional movement disorders, Motor conversion disorder, Functional weakness, Brain connectivity, Functional connectivity, Functional magnetic resonance imaging, Temporoparietal junction, Precuneus, BDI, Beck depression inventory, CON, Controls, DMN, Default mode network, EC, Eigenvector centrality, EC-ADD, EC adding 1 to the correlation matrix, EC-RLC, EC based on rectified linear unit (ReLU) correlation, EPI, Echo-planar imaging, FD, Framewise displacement, FMD, Functional movement disorders, fMRI, Functional magnetic resonance imaging, FW, Functional weakness, FWE, Family-wise error, GCOR, Global correlation, ICC, Intrinsic connectivity, IPL, Inferior parietal lobule, MNI, Montreal neurological institute, MRI, Magnetic resonance imaging, NIfTI, Neuroimaging informatics technology initiative, STAI, State-trait anxiety inventory, SFMDRS, Simplified FMD rating scale, SMA, Supplementary motor area, TPJ, Temporoparietal junction

## Abstract

**Background:**

Functional movement disorders, a common cause of neurological disabilities, can occur with heterogeneous motor manifestations including functional weakness. However, the underlying mechanisms related to brain function and connectivity are unknown.

**Objective:**

To identify brain connectivity alterations related to functional weakness we assessed network centrality changes in a group of patients with heterogeneous motor manifestations using task-free functional MRI in combination with different network centrality approaches.

**Methods:**

Task-free functional MRI was performed in 48 patients with heterogeneous motor manifestations including 28 patients showing functional weakness and 65 age- and sex-matched healthy controls. Functional connectivity differences were assessed using different network centrality approaches, i.e. global correlation, eigenvector centrality, and intrinsic connectivity. Motor symptom severity was assessed using The Simplified Functional Movement Disorders Rating Scale and correlated with network centrality.

**Results:**

Comparing patients with and without functional weakness showed significant network centrality differences in the left temporoparietal junction and precuneus. Patients with functional weakness showed increased centrality in the same anatomical regions when comparing functional weakness with healthy controls. Moreover, in the same regions, patients with functional weakness showed a positive correlation between motor symptom severity and network centrality. This correlation was shown to be specific to functional weakness with an interaction analysis, confirming a significant difference between patients with and without functional weakness.

**Conclusions:**

We identified the temporoparietal junction and precuneus as key regions involved in brain connectivity alterations related to functional weakness. We propose that both regions may be promising targets for phenotype-specific non-invasive brain stimulation.

## Highlights

• Brain connectivity alterations were found in functional movement disorders.
• Hyperconnectivity in temporoparietal junction and precuneus in functional weakness.
• Consistent brain connectivity differences with four different centrality measures.
• Motor symptom severity correlates positively with connectivity in functional weakness.

## 1 Introduction

Functional weakness (FW) is a common motor presentation in functional movement disorders (FMD) that often persists and causes significant disabilities (Stone et al., 2010). It can be present with or without other symptoms such as tremor, dystonia, gait disorders, and myoclonus (Espay et al., 2018a, Stone and Aybek, 2016). Like other FMD symptoms, FW is inconsistent, i.e. characterized by a fluctuation of weakness severity over time and discordant performance between clinical assessment. It is clinically incongruent with any known neurological disease (Espay et al., 2018a) and the underlying neuropathological mechanisms are unclear. Thus, in order to shed more light on potential FW-related alterations of brain function, the aim of the current paper is to identify functional brain connectivity changes related to FW in a group of FMD patients using functional magnetic resonance imaging (fMRI).

Current neurobiological models of FMD symptoms are based on predictive coding of perception and movement control (Edwards and Bhatia, 2012, Van den Bergh et al., 2017). These models suggest that functional symptoms arise from the development of abnormal “priors” or predictions, the expression of which is driven by an abnormal allocation of attention. A key assumption of this proposed mechanism is that the same basic computational phenomenon can account for functional symptoms across motor, sensory, and interoceptive domains (Baizabal-Carvallo et al., 2019, Edwards and Bhatia, 2012). These views have been reflected in a trans-diagnostic approach to find common mechanisms in phenotypically heterogeneous cohorts/groups of patients with functional neurological disorder (Perez et al., 2021). However, only a few studies have aimed at identifying subtype-specific changes, such as differences between mobile and fixed functional dystonia (Canu et al., 2020, Tomic et al., 2020) or between FMD and dissociative seizures (Sojka et al., 2021).

Different aspects of abnormal motor control (e.g. movement conceptualization, intention, or execution), that are assumed to play a role across FMD variants, have only been addressed in a small number of functional imaging studies investigating specific subpopulations of patients (de Lange et al., 2007, Hassa et al., 2016, Maurer et al., 2016, Nahab et al., 2017, van Beilen et al., 2011, Voon et al., 2011, Voon et al., 2010b). Thus, it remains unclear whether the different motor manifestations of FMD have underlying neuroanatomic and neurophysiological commonalities or whether they differ. Neural correlates specific to FW are unknown but could involve brain areas that are implicated in: motor control (i.e. impaired movement initiation or motor inhibition); sensory processing (i.e. abnormal pattern of sensory feedback); or areas involved in top-down, higher-order regulatory processes, perception of self-agency, or other self-referential processes (Baizabal-Carvallo et al., 2019).

FMD with abnormal movements have features of voluntary movements but are perceived as involuntary. In contrast to functional abnormal movements, FW is associated with a reduced range of motion or complete absence of movement despite voluntary effort to execute it (Hallett, 2010). Therefore, we hypothesized that FW may be related to a different self-referential network not directly linked to the sense of agency. In particular, we assumed that the parietal nodes of the default mode network (DMN) could play a role in FW, since this network is involved in the generation of complex internal models of various aspects of self-perception (Igelstrom and Graziano, 2017, Yeshurun et al., 2021). We therefore hypothesized that disruption within the parietal lobe may play a major role in distinguishing FW from no-FW FMD subjects. In order to further investigate the pathophysiological mechanisms of FW, we used task-free fMRI in combination with different network centrality approaches.

The approach of task-free (also often called “resting-state”) fMRI was established in order to investigate correlations between fMRI time courses (Biswal et al., 1995). It was concluded that correlations of low frequency fluctuations and thus correlations between the fMRI signals of different brain regions reflect functional connectivity. Later it was shown that these correlations are altered with ageing (Dennis and Thompson, 2014, Ferreira and Busatto, 2013) and different movement disorders, e.g. in Parkinson’s disease (Mueller et al., 2018, Tahmasian et al., 2015, Wolters et al., 2019), in essential tremor (Li et al., 2021, Mueller et al., 2017, Wang et al., 2018), and also in FMD (Maurer et al., 2016, Nahab et al., 2017). Note that there are various methods for investigating brain connectivity alterations using resting-state fMRI with graph theory approaches (Bassett and Bullmore, 2006, Bullmore and Sporns, 2009, Margulies et al., 2010). The aim of the current study was to detect FW-related alterations within the major hubs of functional brain connectivity. It is known from other movement disorders as e.g. Parkinson’s disease that disease pathology is related to changes within the topography of the hubs of functional brain connectivity which can be assessed by network centrality (Lou et al., 2015, Mueller et al., 2019, Mueller et al., 2017). Therefore, we used various centrality approaches to investigate changes within network hubs relating to FW including the approach of intrinsic connectivity (Martuzzi et al., 2011) but also eigenvector centrality (Lohmann et al., 2010). The comparison between different centrality approaches is very interesting from a methodological point of view as there is a general debate about the reliability of brain connectivity measures with resting-state fMRI (Holiga et al., 2018). We expected similar findings with all network centrality measures as we hypothesize a major effect of FW on the key nodes of functional brain networks.

To assess FW-related brain connectivity alterations, we used different variations of network centrality, namely, global correlation (GCOR) (Whitfield-Gabrieli and Nieto-Castanon, 2012), eigenvector centrality (EC) (Lohmann et al., 2010), and intrinsic connectivity (ICC) (Martuzzi et al., 2011). These network centrality approaches allowed us to describe the importance of network nodes and determine the role of various brain regions within brain networks in FW. Specifically, we analyzed brain connectivity differences between FMD patients with and without FW. In order to characterize centrality changes that are specific to FW and to contextualize findings as inside or outside the normal range, we also assessed centrality differences between patients with FW and healthy controls. Finally, as the evidence for neural correlates of motor symptom severity in FMD is generally lacking, we further searched for a potential relationship between brain connectivity and symptom severity, assessed with the Simplified FMD Rating Scale (SFMDRS) (Nielsen et al., 2017). We hypothesized that FW-specific brain regions would be detected by centrality measurements that would also reflect symptom severity.

## 2 Methods

### 2.1 Participants

Forty-eight patients with clinically definite FMD according to Gupta and Lang criteria (Gupta and Lang, 2009) (37 females, age 45.3 ± 9.7 years, mean ± SD) with heterogeneous motor phenotypes were compared to 65 control subjects (41 females, 46.0 ± 9.4 years, denoted as *CON*). The FMD diagnosis was established following a detailed clinical interview and an examination by an experienced movement disorders specialist (TS) based on positive signs of inconsistence and incongruency with other neurological disorders, also in accordance with the criteria of the Diagnostic and Statistical Manual of Mental disorders (DSM-5) (Apa, 2013, Daum et al., 2014, Edwards and Bhatia, 2012, Espay and Lang, 2015). All patients exhibited non-paroxysmal motor symptoms. For all controls, a complete medical history was obtained and a full neurological examination was performed showing no signs of a neurological disorder. To provide a naturalistic control group that could account for common psychiatric comorbidities found in FMD, controls with clinically salient depression, anxiety, and/or with a current prescription of an antidepressant use were also included. Clinically salient depression and anxiety are defined as presence of symptoms of depression and/or anxiety most of the day affecting most or all activities. Depression and anxiety were assessed with the Beck Depression Inventory (BDI) and the State-Trait Anxiety Inventory (STAI) (Beck et al., 1961, Spielberger, 1983). The demographic data and basic clinical information are provided in Table 1.

**Table 1:** Demographic data of patients and healthy controls*.

	CON	FMD	*P*	FW	no-FW	*P*
						
*N*	65	48		28	20	
female/male^†^	41/24	37/11	0.15	24/4	13/7	0.16
age (years)^+^	46.0 ± 9.4	45.3 ± 9.7	0.68	44.2 ± 8.8	46.7 ± 10.9	0.38
psychotropic drugs yes/no^†^	15/50	21/27	0.025	13/15	8/12	0.77
STAI^+^	39.7 ± 10.9	48.8 ± 13.1	<0.001	49.1 ± 12.1	48.3 ± 14.6	0.85
BDI^+^	8.2 ± 10.0	19.1 ± 13.9	<0.001	19.3 ± 13.9	18.8 ± 14.3	0.91
disease onset (years)^+^		38.6 ± 10.7		37.0 ± 9.0	40.8 ± 12.5	0.23
disease duration (years)^+^		5.6 ± 5.3		6.4 ± 6.2	4.5 ± 3.5	0.22
SFMDRS^+^		12.1 ± 7.7		13.3 ± 7.5	10.4 ± 7.7	0.20

*The table lists demographic data and statistical group comparisons between patients with functional movement disorder (FMD) and healthy controls (CON), and between FMD patients with and without functional weakness (FW and no-FW). STAI − State-trait anxiety inventory; BDI – Beck depression inventory; SFMDRS − Simplified FMD rating scale; ^†^Fisher’s exact test (two-tailed); ^+^Independent samples *t*-test with equal variances (two-tailed).

For each FMD patient we evaluated and classified symptoms as functional weakness (FW) and abnormal movements involving tremor, dystonia, myoclonus, or gait disorder. Thereafter, the group of all FMD patients was then divided into two subgroups; patients with FW (denoted as *FW*) and patients without FW (denoted as *no-FW*, see Table 1 for further details). Patients with abnormal movements and concomitant FW were included in the FW group. For all patients, motor symptom severity was further assessed using the Simplified Functional Movement Disorder Rating Scale (SFMDRS) (Nielsen et al., 2017). The presence or absence of abnormal movement at each of seven body regions was recorded and rated according to symptom severity and duration, along with gait and speech severity and duration (maximum score: 54). Note that the SFMDRS was only published in March 2017 (Nielsen et al., 2017), and therefore, a subset of 15 patients was assessed retrospectively from video recordings of neurological examinations acquired at the inclusion to the study. The video-recordings included a complete neurological examination including assessment of rule-in signs demonstrating inconsistency of abnormal movements and weakness. In all patients who were assessed retrospectively, the medical report describing the complete neurological examination (both positive and negative findings) was reviewed to ensure that all present motor features were documented in the video-recording and rated. All neurological assessments and SFMDRS ratings (including those from video recordings) were performed by the same examiner (TS).

All data were collected between September 2014 and February 2021. On the day of MRI data acquisition all participants also completed STAI (Spielberger, 1983). Concomitant medication with psychotropic effects were recorded in all subjects. Antidepressants are known to affect brain connectivity (McCabe and Mishor, 2011), therefore the intake of psychotropic drugs was taken into account in data analysis (see below). Twenty patients and 15 control subjects were on antidepressant treatment. The study was approved by the ethics committee of the General University Hospital in Prague (approval number 26/15 grant) and all participants gave their written informed consent to participate in the study. All procedures conformed to the Declaration of Helsinki.

### 2.2 Image acquisition

Functional MRI was obtained using a 3-T MAGNETOM Skyra scanner (Siemens Healthineers, Erlangen, Germany) using a 32-channel head array receive coil with the Syngo MR E11 software and a T_2_*-weighted gradient-echo echo-planar imaging (EPI) sequence (repetition time 2 s; echo time 30 ms; flip angle 90°). The following image dimensions were used: acquisition matrix 64×64 pixels, in-plane resolution 3×3 mm^2^, 30 axial slices with a slice thickness of 3 mm (0.45 mm gap), ascending slice order, nominal image resolution 3×3×3.45 mm^3^. For every participant, 304 functional volumes were acquired resulting in a total scanning time of 10 min and 8 s. For all subjects, image acquisition was performed in the so-called “resting-state”. Participants were instructed to fixate on a visual red crosshair, remain still and awake, and not think of anything in particular. Note that all participants were scanned with the same scanning sequence, i.e. the scanning sequence was not changed during the period of data acquisition.

### 2.3 Image pre-processing

All resting-state fMRI data sets were processed using the CONN toolbox rev. 20b (Whitfield-Gabrieli and Nieto-Castanon, 2012) and SPM12 rev. 7771 (Wellcome Centre for Human Neuroimaging, University College London, UK) with Matlab 9.10 R2021a (The MathWorks, Inc.). Pre-processing was performed using the default pipeline within the CONN toolbox including realignment for motion correction and unwarping to correct for EPI distortions (using the SPM’s realign and unwarp module with the six translational and rotational parameters), slice-time correction, and normalization to the Montreal Neurological Institute (MNI) space, based on the unified segmentation approach (Ashburner and Friston, 2005) that includes image co-registration, tissue classification, and bias correction to be combined within the same generative model. Thereafter, spatial filtering was applied using a Gaussian kernel with 8-mm full width at half maximum. Image pre-processing also included denoising that was performed within the CONN toolbox. To correct for nuisance signal fluctuations, a regression analysis was computed using the scan-to-scan changes in global signal and the framewise displacement timeseries (FD) obtained by the CONN toolbox. Note that FD is very sensitive to identify small movements due to the lower floor in the signal (Power et al., 2012). Pre-processing was finalized using detrending and high-pass filtering using 0.015 Hz to achieve a baseline correction.

### 2.4 Centrality group analysis

For each participant global correlation (GCOR) and intrinsic connectivity (ICC) (Martuzzi et al., 2011) was computed within the CONN toolbox (Whitfield-Gabrieli and Nieto-Castanon, 2012). In addition, we computed a further centrality measure, namely eigenvector centrality (EC) (Lohmann et al., 2010), using the Lipsia software (Lohmann et al., 2001). To obtain the EC, a similarity matrix was computed using the correlation coefficient between all fMRI time courses. In order to use a similarity matrix with non-negative elements, we added the number one to all correlations (the ‘ADD’ approach (Wink et al., 2012)) and further used a new correlating metric ‘RLC’ that offers a similarity matrix with non-negative entries (Lohmann et al., 2018). Here, ‘RLC’ stands for ‘ReLU correlation’ based on the Rectified Linear Unit (ReLU) that is widely used in the context of artificial neural networks (Nair and Hinton, 2010). Note that both EC approaches ADD and RLC are programmed in a memory-efficient way and can be used with high resolution imaging data (Lohmann et al., 2018).

As global signal regression might introduce spurious correlations and thus affect our centrality results (Colenbier et al., 2020, Liu et al., 2017), image pre-processing was performed twice with and without nuisance regression, and GCOR was computed with both pipelines.

After computing all four types of network centrality (GCOR, ICC, EC-ADD, EC-RLC), group analyses were performed using SPM12 with the general linear model and a full factorial design implemented with the three groups (FW, no-FW, CON). The use of antidepressant/anxiolytic medication was included as an additional factor. The model also included age, sex, and STAI as additional nuisance covariates. For patients, we also included disease onset and disease duration to control the inhomogeneity within the subgroups of patients. After the parameter estimation, a statistical analysis was performed using *T*-contrasts in order to investigate centrality differences between groups. As we were primarily interested in FW, we computed the contrasts between FW and no-FW, between FW and CON, and between FW and no-FW+ (where *no-FW+* denotes the joint group of participants without FW: no-FW-patients and healthy controls). In addition, we also computed the contrast between no-FW and CON. The resulting statistical parametric maps were assessed using a cluster-defining threshold of *P* < 0.001. Significant clusters were obtained with *P* < 0.05 including a correction for multiple comparisons with family-wise error (FWE) correction at cluster-level (Flandin and Friston, 2019, Friston et al., 1994, Worsley et al., 1996).

In order to further investigate network centrality alterations in terms of functional connectivity between brain regions, seed-based connectivity analysis was performed with the CONN toolbox using seed regions obtained with the GCOR measure and the FW > noFW+ contrast with a threshold of *P* < 0.0001. For each seed-region, seed-based correlation maps were obtained for each subject, and significant FW vs. no-FW+ group differences were detected using the same statistical approach as used with the network centrality analysis.

### 2.5 Simplified FMD rating scale correlations with centrality measures

In addition to group analyses investigating brain connectivity differences between participants with and without FW, we also studied potential correlations between SFMDRS and brain connectivity within both the FW and no-FW groups. To identify a potential group difference with respect to the correlation between SFMDRS and brain connectivity, we assessed the interaction between both factors ‘SFMDRS’ and ‘GROUP’ (FW/no-FW) with all four centrality measures (GCOR, ICC, EC-RLC, and EC-ADD), using the same model as with the group comparisons, including the SFMDRS as a covariate of interest. Here, the SFMDRS covariate was implemented to model an interaction between SFMDRS and GROUP. Subsequent statistical analysis was performed using the same statistical threshold as was used with the group comparisons, i.e. resulting statistical parametric maps were assessed using a cluster-defining threshold of *P* < 0.001, and significant clusters were obtained with *P* < 0.05 using correction for multiple comparisons with family-wise error (FWE) correction at cluster-level (Flandin and Friston, 2019, Friston et al., 1994, Worsley et al., 1996). After the interaction analysis, post-hoc tests were performed within the FW and no-FW groups separately, to assess potential positive or negative correlations between SFMDRS and the four centrality measures.

### 2.6 Motion effects

Due to motion-induced signal fluctuations, head motion can bias the connectivity analysis and resulting connectivity values (Parkes et al., 2018, Satterthwaite et al., 2012). This could be a particular problem if the degree of motion-related artifacts were to vary between patients and controls, or between FW and no-FW patients. Therefore, we checked for differences in head motion between these groups by computing the framewise displacement (FD) calculated as the sum of the absolute values of the differential of the realignment estimates (Power et al., 2012). For input we used the translational and rotational motion parameters obtained by SPM's motion correction. For the whole series of 304 functional images, the motion between volumes was characterised using 303 FD values for each subject. Finally, for each subject, all FD time courses were characterised by the mean FD, the maximum FD, and the number of FD values exceeding 1 mm.

### 2.7 Visualisation

Figures showing orthogonal brain slices were generated using the Mango software v4.1 (Research Imaging Institute, University of Texas Health Science Center at San Antonio) with the ‘Build Surface’ option and the ‘Cut Plane’ feature. Finally, statistical parametric maps were imported using the ‘Add Overlay’ function. Dot-plots and contrast estimates were directly obtained from SPM12.

### 2.8 Data availability

Datasets analyzed during the current study are available on reasonable request. All data will be anonymized. Functional MRI data will be available in pre-processed fashion in the NIfTI format without any personal metadata. All individual brain connectivity maps and all subsequent statistical analyses using SPM12 are publicly available in the Mendeley Data repository “Centrality and seed-based correlation maps obtained with functional MRI” (Mueller et al., 2022) https://doi.org/10.17632/w35fvmtnf2.1.

## 3 Results

### 3.1 Clinical phenotypes

Twenty-eight patients exhibited FW, 15 of them presented with pure FW, and 13 with a mixed phenotype combining FW and other types of FMD (tremor, dystonia, myoclonus, or gait disorder). Twenty patients showed no signs of FW (only positive symptoms of FMD). Thirty patients manifested a combined phenotype (e.g. tremor and dystonia, FW and myoclonus). Between the FW and no-FW groups there were no significant differences in age, sex, STAI, BDI, SFMDRS, disease onset, disease duration, or antidepressant/anxiolytic medication (see Table 1). Note that we found a significant correlation between STAI and BDI in patients (*R* = 0.87, *P* < 0.001) and controls (*R* = 0.83, *P* < 0.001).

### 3.2 Centrality group analysis

**Fig. 1:**
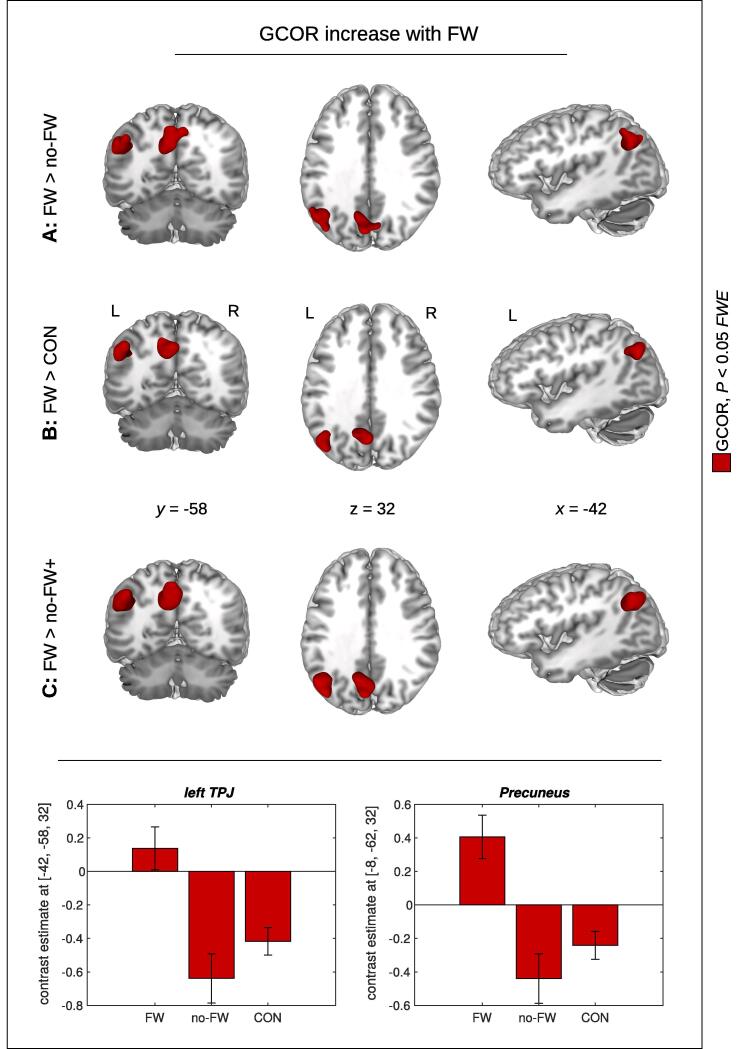
Brain network centrality increase with functional weakness (FW) using global correlation (GCOR). Significant GCOR differences were found in the precuneus and in the left temporoparietal junction (TPJ) with both comparisons between patients with and without FW (**A:** FW > no-FW), and between FW patients and healthy controls (**B:** FW > CON). The same result was obtained when comparing FW patients with all participants showing no FW (**C:** FW > no-FW+ ). Bar plots show contrast estimates for the maximum voxel in the left TPJ and the precuneus. Significant results are shown in red with *P* < 0.05 using family-wise error (FWE) correction at cluster-level (see Table 2 for all 4 centrality measures). *x, y, z* – coordinates in mm; L – left; R – right. (For interpretation of the references to color in this figure legend, the reader is referred to the web version of this article.)

When investigating centrality differences between patients with and without FW, using the contrast FW > no-FW, we obtained very consistent results with all four centrality measures. We obtained a significant cluster in the left temporoparietal junction (TPJ) with GCOR and both measures of EC (Table 2A; Fig. 1A). This result was also obtained with ICC using an uncorrected threshold. In addition to the left TPJ, the comparison FW > no-FW revealed another significant cluster in the precuneus with both GCOR and EC-ADD (Table 2A; Fig. 1A). This result was also obtained with EC-RLC and ICC when using an uncorrected threshold.

**Table 2:** Brain network centrality increase in functional weakness (FW)*.

			cluster-level			peak-level				
			*P_FWE_*	*k*	*P*	*T*	*Z*	*x*	*y*	*z*
**A:** FW > no-FW	TPJ	GCOR	0.011	230	0.001	4.34	4.14	−44	−58	32
		EC-RLC	0.002	327	<0.001	4.77	4.52	−44	−58	32
		EC-ADD	0.004	292	<0.001	4.50	4.28	−44	−58	32
		*ICC*	*0.372*	*67*	*0.048*	*3.64*	*3.52*	*−36*	*−60*	*36*

	Precuneus	GCOR	0.009	238	0.001	4.45	4.24	−8	−62	32
		*EC-RLC*	*0.052*	*157*	*0.006*	*4.51*	*4.30*	*−10*	*−60*	*30*
		EC-ADD	0.024	201	0.003	4.76	4.51	−10	−60	30
*ICC*		*0.189*	*95*	*0.022*	*3.74*	*3.61*	*0*	*−68*	*32*

**B:** FW > CON	TPJ	GCOR	0.036	171	0.004	4.54	4.32	−42	−68	34
		EC-RLC	0.027	189	0.003	4.61	4.38	−42	−66	32
		*EC-ADD*	*0.080*	*141*	*0.009*	*4.34*	*4.14*	*−44*	*−66*	*32*
		ICC	<0.001	618	<0.001	5.66	5.37	−40	−68	34

	Precuneus	GCOR	0.017	207	0.002	4.23	4.05	−14	−56	38
		EC-RLC	0.011	234	0.001	4.10	3.93	−8	−58	36
		EC-ADD	0.015	225	0.002	4.21	4.03	−14	−56	38
ICC		<0.001	625	<0.001	4.70	4.46	0	−56	36

**C:** FW > no-FW+	TPJ	GCOR	0.002	330	<0.001	4.59	4.37	−42	−58	32
		EC-RLC	<0.001	433	<0.001	4.84	4.58	−44	−58	32
		EC-ADD	0.001	372	<0.001	4.59	4.37	−42	−58	32
		ICC	<0.001	471	<0.001	4.65	4.42	−40	−70	34

	Precuneus	GCOR	0.001	355	<0.001	4.84	4.58	−8	−62	32
		EC-RLC	0.003	311	<0.001	4.60	4.37	−10	−60	30
		EC-ADD	0.003	323	<0.001	4.86	4.60	−8	−62	32
ICC		<0.001	632	<0.001	4.35	4.16	0	−64	30

*(**A:**) Comparing between patients with and without functional weakness (**A:** FW > no-FW), we obtained increased brain network centrality in the left temporoparietal junction (TPJ) and the precuneus with global correlation (GCOR) and eigenvector centrality (EC-RLC and EC-ADD, respectively). Intrinsic connectivity (ICC) showed centrality differences in the same regions with an uncorrected threshold (shown in italics). (**B:**) Comparing FW patients with healthy controls (**B:** FW > CON) revealed the same centrality differences as obtained with comparison to the no-FW patients, however, in contrast to (**A:**), significant results were also obtained with ICC. (**C:**) Comparing FW patients with all participants showing no FW (**C:** FW > no-FW+) showed again the same centrality differences obtained with both comparisons (**A:**) and (**B:**). Significant differences were obtained with *P* < 0.05 using family-wise error (FWE) correction at cluster-level. Non-significant differences are shown in italics. *P_FWE_* – *P*-value after FWE correction at cluster-level; *k* – size of cluster in voxels; *x, y, z* – coordinates in mm.

**Fig. 2:**
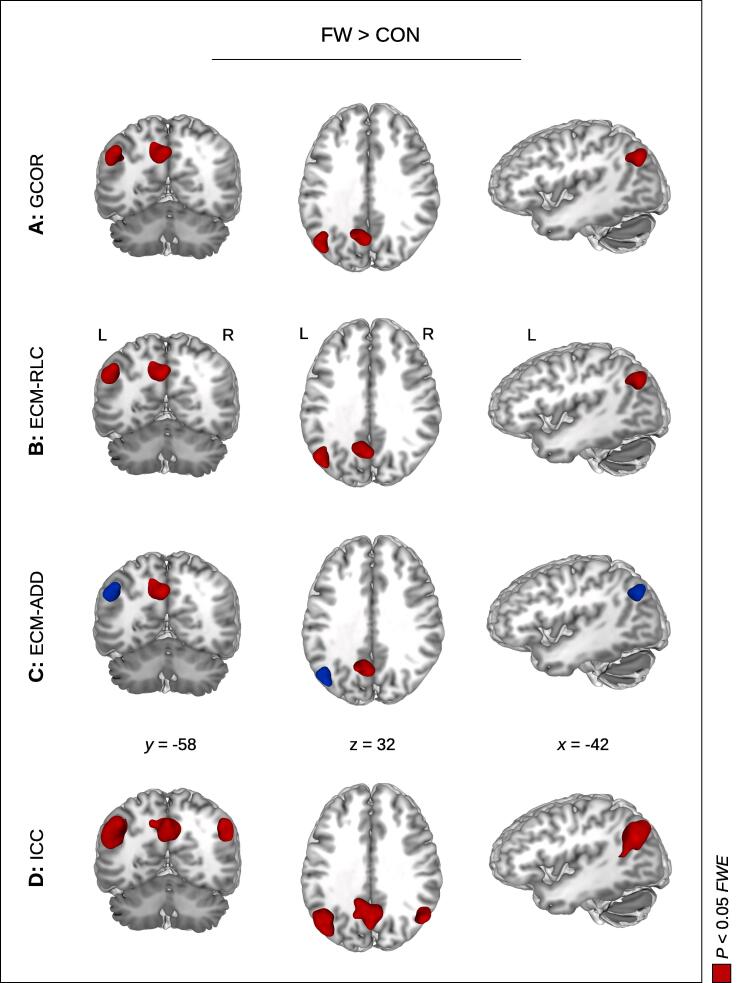
Brain network centrality increase in functional weakness (FW) compared to healthy controls (CON). Significant centrality alterations were found in the precuneus and the left temporoparietal junction (TPJ) with all four centrality measures: global correlation (**A:** GCOR), intrinsic connectivity (**D:** ICC), and both measures of eigenvector centrality with two different approaches handling the negative correlations (**B:** EC-RLC and **C:** EC-ADD, respectively). Note that the left and the right TPJ was found with ICC. Significant results are shown in red with *P* < 0.05 using family-wise error (FWE) correction at cluster-level (see also Table 2). The blue color shows the result with EC-ADD using an uncorrected threshold. *x, y, z* – coordinates in mm; L – left; R – right. (For interpretation of the references to color in this figure legend, the reader is referred to the web version of this article.)

Looking at the contrast FW > CON, we again obtained a significant centrality difference in the left TPJ with GCOR, EC-RLC, and ICC (Table 2B; Fig. 1B; Fig. 2), and with EC-ADD using an uncorrected threshold (Fig. 2C). We also obtained a significant centrality difference in the precuneus with all four centrality measures. Thus, we found the same anatomical brain regions with both comparisons FW > no-FW (Table 2A; Fig. 1A) and FW > CON (Table 2B; Fig. 1B). Looking at FW > no-FW+ using the extended no-FW+ group including all participants from both groups no-FW and CON, we received a robust finding in the left TPJ and in the precuneus with all four centrality measures (Table 2C; Fig. 1C).

Investigating GCOR with and without nuisance regression showed only subtle differences between both analyses. Skipping the regression analysis during pre-processing, we received the same FW-related GCOR increase using the contrasts FW > no-FW, FW > CON, and FW > no-FW+ (compare Fig. 1 and Supplementary Figure S1).

**Fig. 3:**
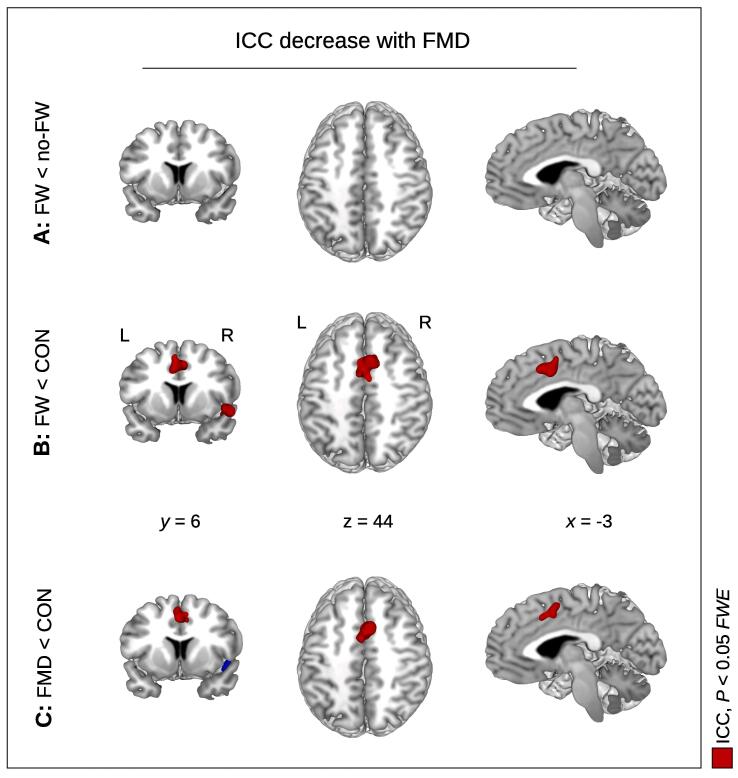
Decreased brain network centrality in functional movement disorder (FMD). (**A:**) Comparing patients with and without functional weakness (FW), we did not obtain any significant centrality decrease with all centrality measures. (**B:**) Comparing FW patients with healthy controls (CON), a diminished intrinsic connectivity (ICC) was found in the supplementary motor area (SMA) and insula. (**C:**) Comparing all FMD patients with healthy controls, the same pattern of centrality decrease was obtained as shown in (**B:**), however, the insula was only found using an uncorrected threshold (shown in blue). Significant results are shown in red with *P* < 0.05 using family-wise error (FWE) correction at cluster-level (see Table 3 for all 4 centrality measures). *x, y, z* – coordinates in mm; L – left; R – right. (For interpretation of the references to color in this figure legend, the reader is referred to the web version of this article.)

We also looked at the inverse contrasts relating to a diminished network centrality in FW compared to the other groups, however, the contrast FW < no-FW did not show any significant centrality results. However, the comparison FW < CON showed a significant centrality decrease in the supplementary motor area (SMA) with ICC and both measures of EC (Table 3A; Fig. 3B). The same region was found with GCOR using an uncorrected threshold. Moreover, we also found a significant cluster in the right insula with all four centrality measures (Table 3A; Fig. 3B). Note that neither cluster (the SMA and the right insula) showed up for the FW < no-FW contrast with any of the centrality measures (Fig. 3A). Which raises the question of whether these findings really relate to FW.

**Table 3:** Brain network centrality decrease in functional movement disorders*.

			cluster-level			peak-level				
			*P_FWE_*	*k*	*P*	*T*	*Z*	*x*	*y*	*z*
**A: FW < CON**	SMA	*GCOR*	*0.067*	*142*	*0.007*	*4.17*	*4.00*	*6*	*14*	*46*
		EC-RLC	0.004	290	<0.001	4.65	4.42	6	14	46
		EC-ADD	0.037	178	0.004	4.40	4.20	6	14	46
		ICC	<0.001	397	<0.001	4.78	4.53	−2	14	40

	Insula	GCOR	0.008	246	0.001	4.06	3.90	52	6	−12
		EC-RLC	0.010	239	0.001	4.24	4.06	52	14	−12
		EC-ADD	0.025	197	0.003	3.95	3.80	46	20	−10
ICC		0.004	271	<0.001	4.32	4.13	50	4	−6

**B: FMD < CON**	SMA	GCOR	0.013	220	0.001	4.26	4.07	8	−8	36
		*EC-RLC*	*0.305*	*78*	*0.039*	*4.20*	*4.02*	*6*	*12*	*46*
		*EC-ADD*	*0.331*	*76*	*0.045*	*3.93*	*3.78*	*8*	*12*	*46*
		ICC	0.007	244	0.001	4.60	4.37	6	12	46

	*Insula*	*GCOR*	*0.113*	*119*	*0.013*	*4.05*	*3.89*	*46*	*18*	*−8*
		*EC-RLC*	*0.211*	*94*	*0.026*	*4.34*	*4.15*	*46*	*18*	*−8*
		*EC-ADD*	*0.310*	*79*	*0.042*	*4.15*	*3.98*	*46*	*18*	*−6*
*ICC*		*0.068*	*138*	*0.007*	*4.36*	*4.16*	*54*	*18*	*0*

*(**A:**) Comparing FW patients with healthy controls (**A:** FW < CON), a significant brain network centrality decrease was obtained in the insula and in the supplementary motor area (SMA) with both measures of eigenvector centrality (EC-RLC and EC-ADD, respectively) and with intrinsic connectivity (ICC). Global correlation (GCOR) also showed a significant centrality decrease in the insula, however, the SMA was only found with an uncorrected threshold (shown in italics). (**B:**) The comparison between all patients with functional movement disorder (FMD) and healthy controls (**B:** FMD < CON) revealed a significant centrality decrease in the SMA using the centrality measures of global correlation (GCOR) and intrinsic connectivity (ICC). The other results were obtained without correction for multiple comparisons (see lines in italics). *P_FWE_* – *P*-value after family-wise error (FWE) correction at cluster-level; *k* – size of cluster in voxels; *x, y, z* – coordinates in mm.

In order to further elaborate a potential diminished centrality in FMD, we looked at the comparison between all FMD patients and healthy controls, i.e. FMD < CON. Interestingly, this comparison showed centrality differences in the same anatomical brain regions as obtained with FW < CON: The comparison FMD < CON showed the same cluster in the SMA obtained using GCOR and ICC (Table 3B; Fig. 3C), and with both EC measures at an uncorrected level. Further, the comparison FMD < CON also revealed the right insula using an uncorrected level. Thus, both comparisons FMD < CON and FW < CON led to the same findings, and no results were obtained with FW < no-FW.

Albeit the primary goal of this study was aimed at FW and the comparison between FW with both groups no-FW and CON, we also looked at potential centrality differences between no-FW and CON. Investigating the contrast no-FW > CON, we did not find any significant result with all four centrality measures. The inverse contrast no-FW < CON showed a significant difference in the posterior cingulate cortex with the centrality measures GCOR and EC-ADD (see Supplementary Figure S2). The same cluster was found with EC-RLC without FWE-correction, but no cluster was found with ICC even when using an uncorrected threshold.

In addition to network centrality group differences described above, we also investigated seed-based correlation using the left TPJ and the precuneus as seed regions. Investigating seed-based correlation maps with the FW > no-FW+ contrast, we obtained an FW-related connectivity increase between both seeds and the middle temporal gyrus (MTG) (Supplementary Figure S3). With the left TPJ as seed region, we also found an FW-related connectivity increase between left TPJ and precuneus, and between left TPJ and left cerebellum. Note that we did not obtain any significant functional connectivity decrease (using the FW < no-FW+ contrast) with both seed-regions.

### 3.3 Simplified FMD rating scale correlations

**Fig. 4:**
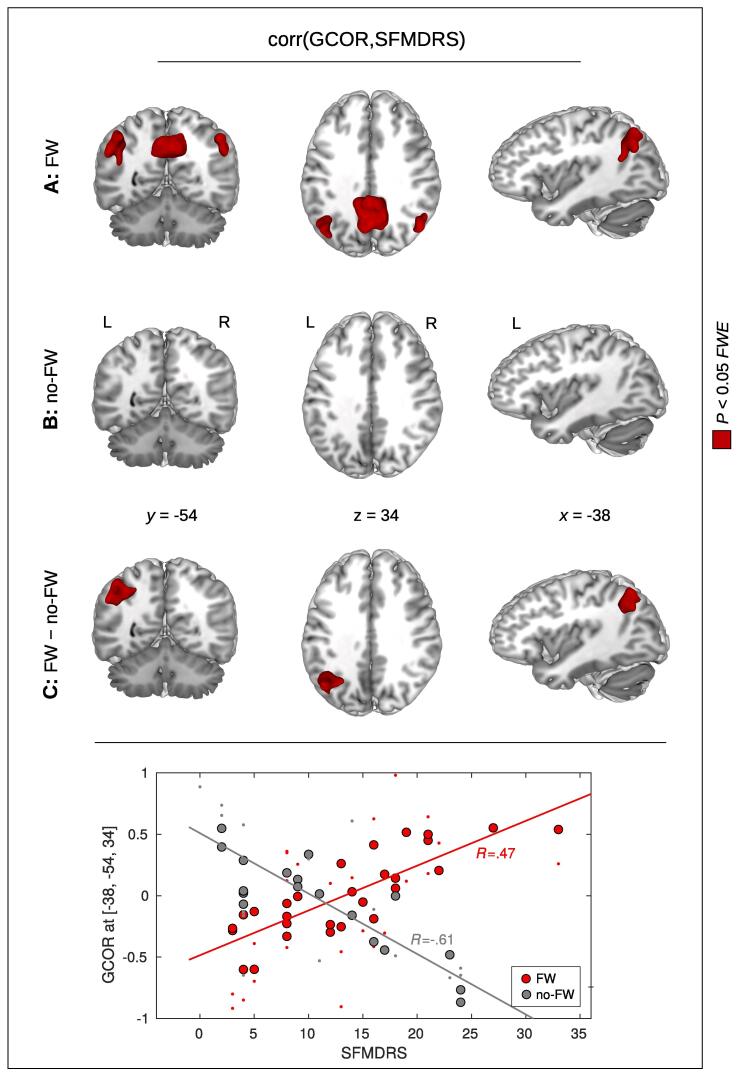
Positive relationship between global correlation (GCOR) and the Simplified Functional Movement Disorder Rating Scale (SFMDRS) in patients with functional weakness (FW). (**A:**) Within the group of FW patients, a significant positive correlation between GCOR and SFMDRS was obtained in the precuneus and in the left and right temporoparietal junction (TPJ). (**B:**) No significant correlation was obtained in the group of patients showing no FW (no-FW). (**C:**) A significant interaction between the factors GROUP (FW/no-FW) and SFMDRS was found in the left TPJ. Significant results are shown in red with *P* < 0.05 using family-wise error (FWE) correction at cluster-level (see Table 4 for all 4 centrality measures). *x, y, z* – coordinates in mm; L – left; R – right. The dot-plot on the bottom shows the GCOR-values in the left TPJ for the FW group (in red) and for the no-FW group (in gray). The bigger dots show the fitted GCOR values within the statistical model while the smaller dots show the zero-mean GCOR values. (For interpretation of the references to color in this figure legend, the reader is referred to the web version of this article.)

To investigate the relationship between brain connectivity and the clinical severity of the FW patients, we studied potential correlations between SFMDRS and network centrality across the whole brain. To test for potential group differences (FW vs. no-FW) with respect to a correlation between SFMDRS and brain network centrality, we used a GLM implementing the interaction between the factors SFMDRS and GROUP (FW/no-FW). Here, we obtained a significant result in the left TPJ using GCOR and both measures of EC (Table 4B; Fig. 4C). We also obtained this result with ICC when using an uncorrected threshold.

**Table 4:** Positive correlation between Simplified Functional Movement Disorders Rating Scale (SFMDRS) and brain network centrality in patients with functional weakness (FW)*.

			cluster-level			peak-level				
			*P_FWE_*	*k*	*P*	*T*	*Z*	*x*	*y*	*z*
**A:** Correlation in FW	TPJ	GCOR	0.005	237	<0.001	4.75	4.14	−46	−62	30
		EC-RLC	0.005	242	<0.001	4.78	4.19	−38	−68	40
		EC-ADD	0.005	253	<0.001	4.57	4.05	−42	−60	32
		*ICC*	*0.160*	*86*	*0.014*	*4.39*	*3.91*	*−40*	*−52*	*20*

	Precuneus	GCOR	<0.001	1328	<0.001	5.29	4.53	10	−54	38
		EC-RLC	<0.001	1119	<0.001	6.31	5.17	12	−56	40
		EC-ADD	<0.001	1188	<0.001	5.31	4.55	14	−54	40
ICC		<0.001	490	<0.001	4.94	4.30	14	−56	40

**B:** Interaction	TPJ	GCOR	0.002	277	<0.001	5.08	4.39	−38	−54	34
		EC-RLC	0.008	223	<0.001	5.27	4.52	−38	−54	34
		EC-ADD	0.009	227	<0.001	5.34	4.57	−38	−54	34
		*ICC*	*0.091*	*105*	*0.008*	*4.32*	*3.86*	*−38*	*−60*	*38*

*(**A:**) Within the group of FW patients, a significant positive correlation between SFMDRS and brain connectivity was observed with global correlation (GCOR) and both measures of eigenvector centrality (EC-RLC and EC-ADD, respectively). Consistently with all 3 approaches, significant positive correlation was detected in the left temporoparietal junction (TPJ), and in the precuneus. Intrinsic connectivity (ICC) showed this positive correlation in the same regions, however, the left TPJ was only found with an uncorrected threshold (see line in italics). (**B:**) A significant interaction between the factors GROUP (FW/no-FW) and SFMDRS was found in the left TPJ with the centrality measures GCOR, EC-RLC, and EC-ADD. Using ICC, this interaction was only obtained with an uncorrected threshold (see line in italics). Significant results were obtained with *P* < 0.05 using family-wise error (FWE) correction at cluster-level. *P_FWE_* – *P*-value after FWE correction at cluster-level; *k* – size of cluster in voxels; *x, y, z* – coordinates in mm.

To further investigate which group was driving the obtained interaction, post-hoc tests were performed to investigate a potential positive or negative correlation between SFMDRS and network centrality within each group separately. Within the group of FW patients, we observed a significant positive correlation between SFMDRS and network centrality in the left TPJ with GCOR and both eigenvector centrality measures EC-RLC and EC-ADD (Table 4A; Fig. 4A). This result was also obtained with ICC using an uncorrected threshold. For the FW patients, we also obtained a significant positive correlation in the precuneus with all four centrality measures (Table 4A; Fig. 4A). Note that this correlation analysis revealed the same cluster pattern as obtained with the group comparison FW > no-FW (left TPJ, precuneus), and thus, FW patients with increased SFMDRS notably contribute to the group difference found in the FW > no-FW comparison. Note that, within the group of FW patients, we did not find any significant negative correlation between SFMDRS and network centrality with any of the four centrality measures.

In the no-FW group, we did not obtain any significant positive correlations between SFMDRS and network centrality with any of the four centrality measures (see Fig. 4B). Note that we did obtain a significant cluster showing a negative correlation between SFMDRS and network centrality with GCOR and both measures of EC, however, this cluster was located in the primary visual cortex. No significant negative correlations were obtained in the TPJ and/or precuneus.

To investigate the effect of nuisance regression to the relationship between SFMDRS and network centrality, correlation analysis was performed twice with GCOR obtained with and without nuisance regression. Skipping nuisance regression during pre-processing does not affect the results as we again obtained a significant positive correlation between SFMDRS and GCOR in the FW group (compare Fig. 4A and Supplementary Figure S4A). We also obtained a significant interaction between the factors SFMDRS and GROUP (FW/no-FW) showing that the positive GCOR-SFMDRS-correlation is specific to FW (compare Fig. 4C and Supplementary Figure S4C).

### 3.4 Motion effects

The analysis of head motion during MR scanning yielded overall very subtle effects. The mean FD was below 0.5 mm for all patients and control subjects, with the exception of two no-FW patients showing a mean FD of 0.57 mm and 0.77 mm, respectively. The maximum FD was less than the nominal voxel size of 3 mm except in a single patient that showed a maximum FD of 3.1 mm. Only 210 out of 34,352 frames from the entire study (i.e. 113 patients × 304 image volumes) indicated single head movements of >1 mm, corresponding to 0.61%. Most importantly, there were no significant FD differences between patients and controls (*P* > 0.3 for both mean and maximum FD), and no significant FD differences between the subgroups of FW and no-FW patients (*P* > 0.2 for both mean and maximum FD).

## 4 Discussion

In this study, we used different centrality approaches to detect significant alterations in functional brain connectivity within functional networks specific to FW in a heterogeneous group of FMD patients. Our major findings were:.

1. With all four different centrality measures, we consistently found an increased interconnectedness of both the left TPJ and the precuneus in FW patients when compared to the no-FW group, and when compared to the group of controls.
2. When comparing FW and no-FW patients, we did not find any centrality decrease with FW. However, comparing FW patients with healthy controls revealed a brain network centrality decrease in the insula and in the SMA. Interestingly, a similar pattern was found when comparing all FMD patients with healthy controls.
3. Within the group of FW patients, a significant positive correlation between SFMDRS and TPJ centrality was observed with global correlation (GCOR) and both measures of eigenvector centrality. Importantly, this correlation was not found in no-FW group, and a significant group difference (i.e. an interaction between the factors GROUP and SFMDRS) was found in the left TPJ.

Using a task-free paradigm and whole-brain analysis, this study provides evidence for the involvement of the TPJ and the precuneus in the context of FW as a specific motor manifestation of FMD. The posterior part of the TPJ and the precuneus are higher-order association cortices which have been hypothesized to be dysfunctional in FMD (Baizabal-Carvallo et al., 2019). Importantly, both regions are also part of the DMN (Buckner and DiNicola, 2019). Thus, in agreement with our hypothesis, our findings suggest that hyperconnectivity of the posterior parietal or the DMN regions may constitute a biomarker of FW. Additional confirmation of the DMN significance in the FW pathophysiology also comes from our seed-based correlations (see Supplementary Figure S3) showing increased connectivity within the areas of this network. In line with this observation, a previous resting-state fMRI study reported an increased functional connectivity strength only in the DMN in patients with pure FW compared to controls (Monsa et al., 2018). However, further (including motor task-based fMRI) studies comparing pure FW with other subtypes of FMD will be necessary for confirmation on whether hyperconnectivity of these posterior parietal regions is a specific fingerprint of FW.

A large body of evidence in literature further supports the role of the TPJ and precuneus in the FMD pathophysiology. Previous fMRI studies using different paradigms in FMD patients have shown abnormal activity and functional connectivity in the TPJ and anatomically and functionally overlapping regions within the supramarginal gyrus, the angular gyrus, or the inferior parietal lobule (IPL) (Aybek et al., 2014, Baek et al., 2017, de Lange et al., 2010, Diez et al., 2019, Hassa et al., 2017, Igelstrom and Graziano, 2017, Schrag et al., 2013, van Beilen et al., 2010, van der Kruijs et al., 2012, Voon et al., 2010b, Wegrzyk et al., 2018). Several of those studies have specifically implicated the right TPJ in abnormal self-agency in FMD patients (Baek et al., 2017, Maurer et al., 2016, Nahab et al., 2017, Voon et al., 2010b). In our study, we found an involvement of the TPJ in the pathophysiology of FW predominantly in the left hemisphere. While the right TPJ/IPL is a key structure in the self-agency network and in the right-lateralized ventral attentional control network implicated in stimulus-driven reorienting of spatial attention (Corbetta and Shulman, 2002, Nahab et al., 2011), the left TPJ/IPL has strong connectivity with the executive control network and is pivotal for configuring non-spatial and motor attention or control processes related to attention (Mengotti et al., 2020). Thus, our finding of increased connectivity of the left TPJ is consistent with our hypothesis that FW is not directly linked to the sense of agency. However, purely attentional mechanisms seem to be unlikely, given that both abnormal movements and FW present with clinically similar attentional effects such as distractibility. Bilateral TPJ seems has been implicated in updating and adjustments of top-down predictions (Geng and Vossel, 2013) suggesting that these processes may be specifically involved in FW.

Involvement of the precuneus was also reported in patients with various functional neurological disorders including FMD in studies using different motor or emotion task-based fMRI (Espay et al., 2018b, Hassa et al., 2017, Sojka et al., 2019, Stone et al., 2007). It has also been reported in studies on self-agency in FMD patients (Baek et al., 2017, Nahab et al., 2017, Voon et al., 2010b) and resting state studies in various populations (Maurer et al., 2016) where the changes in activation or functional connectivity also involved the TPJ. Recent work has also investigated connectivity in FMD and has shown alterations including dynamic changes of functional brain connectivity in the precuneus and posterior midline (Marapin et al., 2021, Marapin et al., 2020).

The current neurobiological model of FMD is based on predictive coding (Edwards and Bhatia, 2012, Van den Bergh et al., 2017). This influential concept posits that the brain's network architecture is an active inference generator that works according to the Bayesian approach to probability via a multilevel neuronal cascade. Learned beliefs about the world and about oneself act as top-down predictions explaining sensory signals that pass prediction errors up the neuronal hierarchy (Friston, 2010). It has also been proposed that the top-down dynamics of generative models detached from sensory or task-specific signals is closely related to the spontaneous activity in brain networks during resting state (Pezzulo et al., 2021). Importantly, the DMN is thought to lie at the top of this processing hierarchy involved in generating and retrieving the most complex and context-dependent schemas of various aspects of the self and the external environment (Friston, 2010, Igelstrom and Graziano, 2017, Yeshurun et al., 2021). The predictive coding account of FMD by Edwards and colleagues (Edwards et al., 2012) proposed that abnormal proprioceptive predictions related to the dynamics of movement are formed within an intermediate motor area (e.g. the SMA) and are afforded too much precision via misdirected attentional gain from higher hierarchical levels. The signal is propagated down the motor hierarchy, producing a proprioceptive prediction error peripherally that is fulfilled by movement or lack of movement in FW. Prediction errors in reporting the unpredicted content of that movement to higher cortical areas (e.g., pre-SMA) are explained in terms of a symptomatic interpretation as involuntary movements or as failure to realize the movement that was intended in FW (Edwards et al., 2012). In our study, however, we found differences between FW and no-FW subjects in the left TPJ and the precuneus that are not directly involved in motor control. Therefore, in agreement with our hypothesis, it is conceivable that hyperconnectivity in these regions that are part of the DMN may reflect excessively strong or dysfunctional priors or schemas related to the body and the sense of the inability to move in FW.

Interestingly, the comparison between FW and no-FW patients showed larger contrast differences than the comparison between FW patients and healthy controls. This connectivity pattern with normal subjects’ connectivity being in the middle between FW and no-FW (see contrast estimates in Fig. 1) seems also to favor reflection of abnormal predictions formation rather than attentional processes or self-agency abnormalities which have been mostly associated with the right TPJ (Baek et al., 2017, Maurer et al., 2016, Nahab et al., 2011, Voon et al., 2010b).

In our study, the role of the bilateral TPJ and the precuneus in FW pathophysiology is further supported by a complementary, within-subgroup approach which found a positive correlation between motor symptom severity and centrality and general connectivity measures in these regions only in FW patients, but not the no-FW group. Note that only one of the previous fMRI studies identified correlates of objectively assessed functional motor symptom severity in a small sample of FMD patients (Faul et al., 2020).

Comparing patients with and without FW using all different centrality measures, we did not find any significant FW-related centrality decrease. Comparing FW patients with healthy controls, we obtained a centrality decrease in the insula and in the SMA, however, due to the absence of this result in the comparison within patients, it is unclear whether this finding can be specifically related to FW. This lack of significant difference might be due to insufficient power caused by a small sample size. Moreover, we found a centrality decrease in the same anatomical regions (insula and SMA) when comparing all FMD patients to healthy controls indicating that this decrease might not be specific to FW, and rather related more to FMD then to FW. That would be also in line with recent findings showing an involvement of the insula within multimodal integration, interoception processing, and self-agency that have been previously shown in different populations of FMD patients (Maurer et al., 2016, Perez et al., 2017, Stone et al., 2007, Voon et al., 2011). The SMA is a key structure in voluntary movement initiation and its hypoactivity was previously reported in numerous studies (Aybek et al., 2015, Kozlowska et al., 2017, Maurer et al., 2016, Voon et al., 2010a, Voon et al., 2011). Our data thus replicated these findings in a larger and more heterogeneous sample of FMD patients and suggests that SMA involvement is rather less phenotype specific.

The finding of increased connectivity of the left TPJ and the precuneus associated with the presence and severity of FW has important clinical implications and provides potential for new treatment approaches. Non-invasive brain stimulation techniques such as repetitive transcranial magnetic stimulation (rTMS) and transcranial direct current stimulation (tDCS) have been applied over the TPJ/parietal cortex for treatment of different conditions including auditory hallucination, tinnitus, and depersonalization (Donaldson et al., 2015). It has also been shown that subjective and behavioral responses to self-referential tasks can be modulated through TMS and tDCS (Bao et al., 2021). Recent experiments with rTMS over the right TPJ have also shown that the self-agency network was amenable to neuromodulation in heathy participants and suggested that manipulation of impaired self-agency in FMD could be used as part of treatment (Zito et al., 2020). We propose that different non-invasive brain stimulation protocols should be tested based on the phenotypical classification and modulation of both the left and the right TPJ and should be studied in interventional trials. Specifically, the effects of inhibitory protocols using cathodal tDCS (Inukai et al., 2016) or lower frequency rTMS (Chen et al., 1997) should be addressed in patients with FW. However, a relative imbalance of TPJ activity between the two hemispheres, which was previously reported in functional dystonia (Schrag et al., 2013), might also play a role in the pathophysiology of motor FMD as it was suggested for neglect syndrome (Mengotti et al., 2020). Carefully selected tasks, individualized neuronavigation, and more targeted and focal TPJ stimulation should be a priority in future studies. Using neurophysiological or imaging techniques either concurrently or pre-post stimulation will help to assess broader distributed effects of non-invasive brain stimulation (Dalong et al., 2021) along with behavioral outcomes.

Note that there are various limitations of this study: The comorbidity of FW and abnormal movements did not allow for a direct comparison of more homogeneous subgroups. Further, the subjects did not undergo a standardized psychiatric examination/structured interview, which would allow for control of a potential bias resulting from different psychiatric comorbidities. The abnormities identified in this study may be disease related, compensatory, or the consequence of differences in unidentified predisposing vulnerabilities and comorbidities. A major limitation is the small sample size. The group of no-FW patients had only a sample size of 20 patients, and two of the no-FW patients showed subtle head movements during MRI data acquisition. Note that the method itself using “resting-state” fMRI has its own drawbacks. Therefore, apart from this technique, future studies should also investigate FW-related connectivity changes using a suitable motor task using the approach of psychophysiological interaction (Friston et al., 1997) using TPJ and precuneus as seed-regions.

## 5 Conclusions

In this fMRI study comparing patients with and without FW, we identified an FW-associated increase of functional connectivity in the left TPJ and the precuneus. Further, these increases correlated with motor symptom severity. The TPJ and the precuneus are important nodes of the multisensory integration network and are known to be involved in self-referential processing, including self-agency and monitoring of one’s own performance, the integration of top-down attentional control with bottom-up processing, and adjustments of top-down predictions. Consistent with predictive coding accounts of FMD, our findings suggest that alterations in these mechanisms might underly different motor phenotypes such as FW. Our findings have important implications for new treatment approaches. Future interventional trials using non-invasive brain stimulation techniques should consider a phenotype-specific pattern of functional connectivity. Specifically, protocols inducing inhibition of the left parietal cortex should be studied in presence of FW.

### CRediT authorship contribution statement

**Karsten Mueller:** Conceptualization, Methodology, Software, Investigation, Writing – review & editing, Formal analysis, Writing – original draft, Visualization. **Filip Růžička:** Conceptualization, Methodology, Software, Investigation, Writing – review & editing, Formal analysis, Writing – original draft, Data curation, Project administration, Resources. **Matěj Slovák:** Conceptualization, Investigation. **Zuzana Forejtová:** Conceptualization, Investigation. **Petr Dušek:** Conceptualization, Investigation, Writing – review & editing. **Pavel Dušek:** Conceptualization. **Robert Jech:** Conceptualization, Methodology, Investigation, Writing – review & editing, Funding acquisition, Supervision, Writing – original draft, Project administration, Resources. **Tereza Serranová:** Conceptualization, Methodology, Investigation, Writing – review & editing, Funding acquisition, Writing – original draft, Data curation, Project administration, Resources.

## Declaration of Competing Interest

The authors declare that they have no known competing financial interests or personal relationships that could have appeared to influence the work reported in this paper.
