# Supplementary figures and images for: Symptom-severity-related brain connectivity alterations in functional movement disorders
Source: Neuroimage Clin. 2022 Mar 3;34:102981. doi: 10.1016/j.nicl.2022.102981 (PMC8921488; doi:10.1016/j.nicl.2022.102981)

GCOR increase with FW

**A:** FW > no-FW

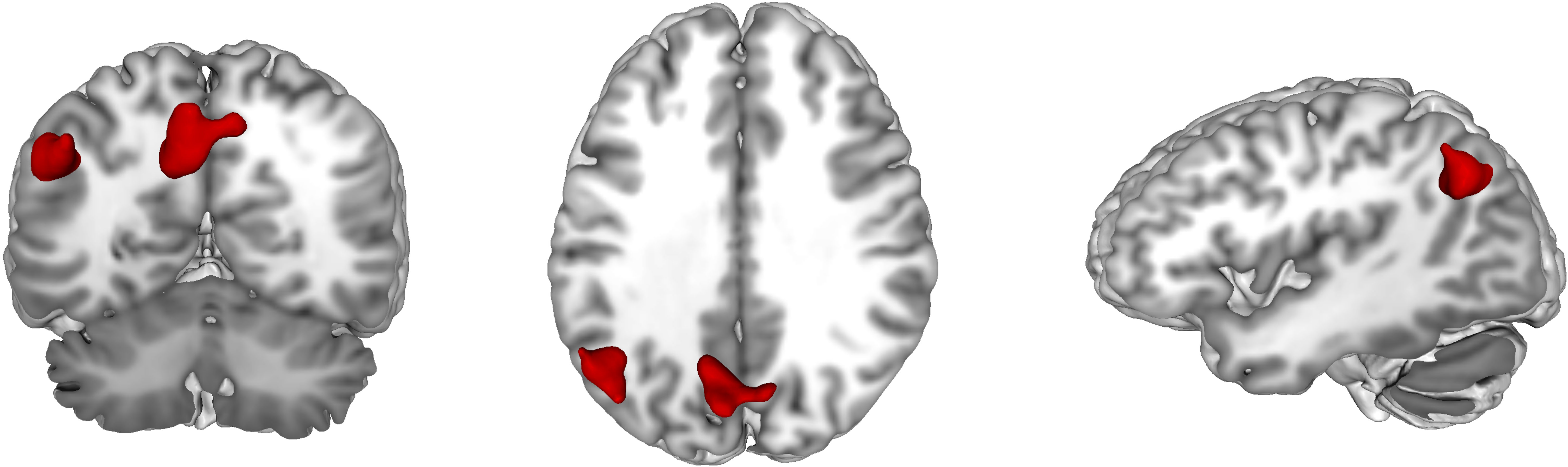

**B:** FW > CON

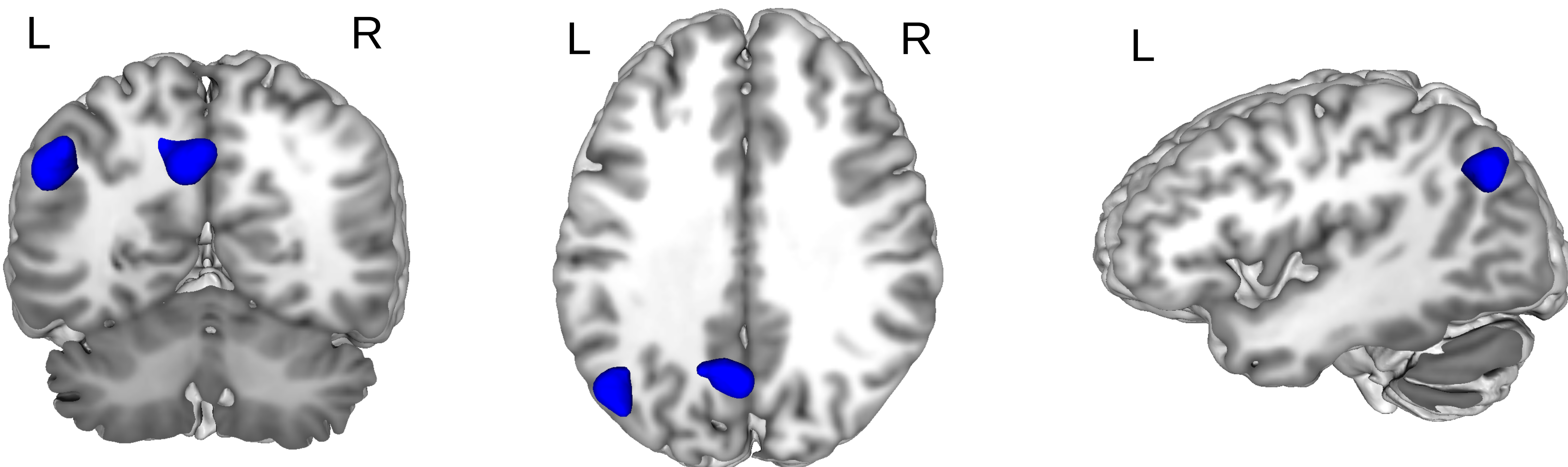

**C:** FW > no-FW+

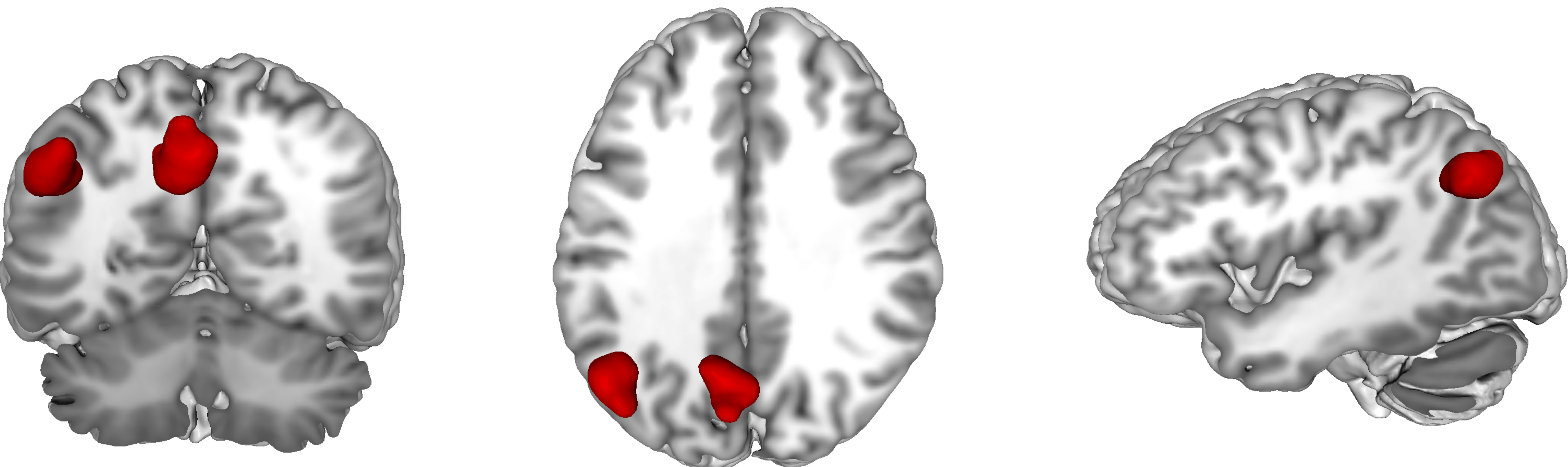

GCOR,  $P < 0.05$  FWE

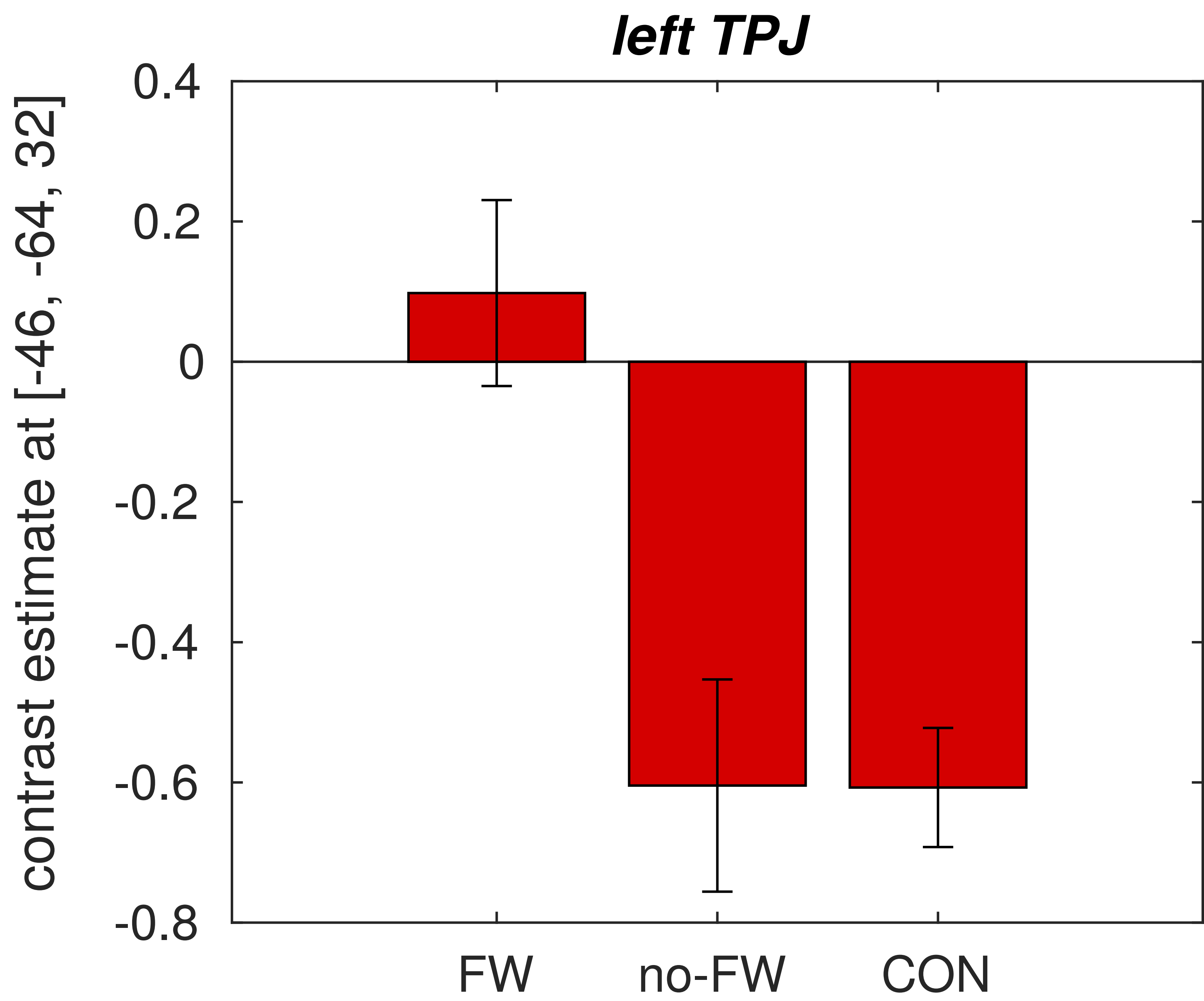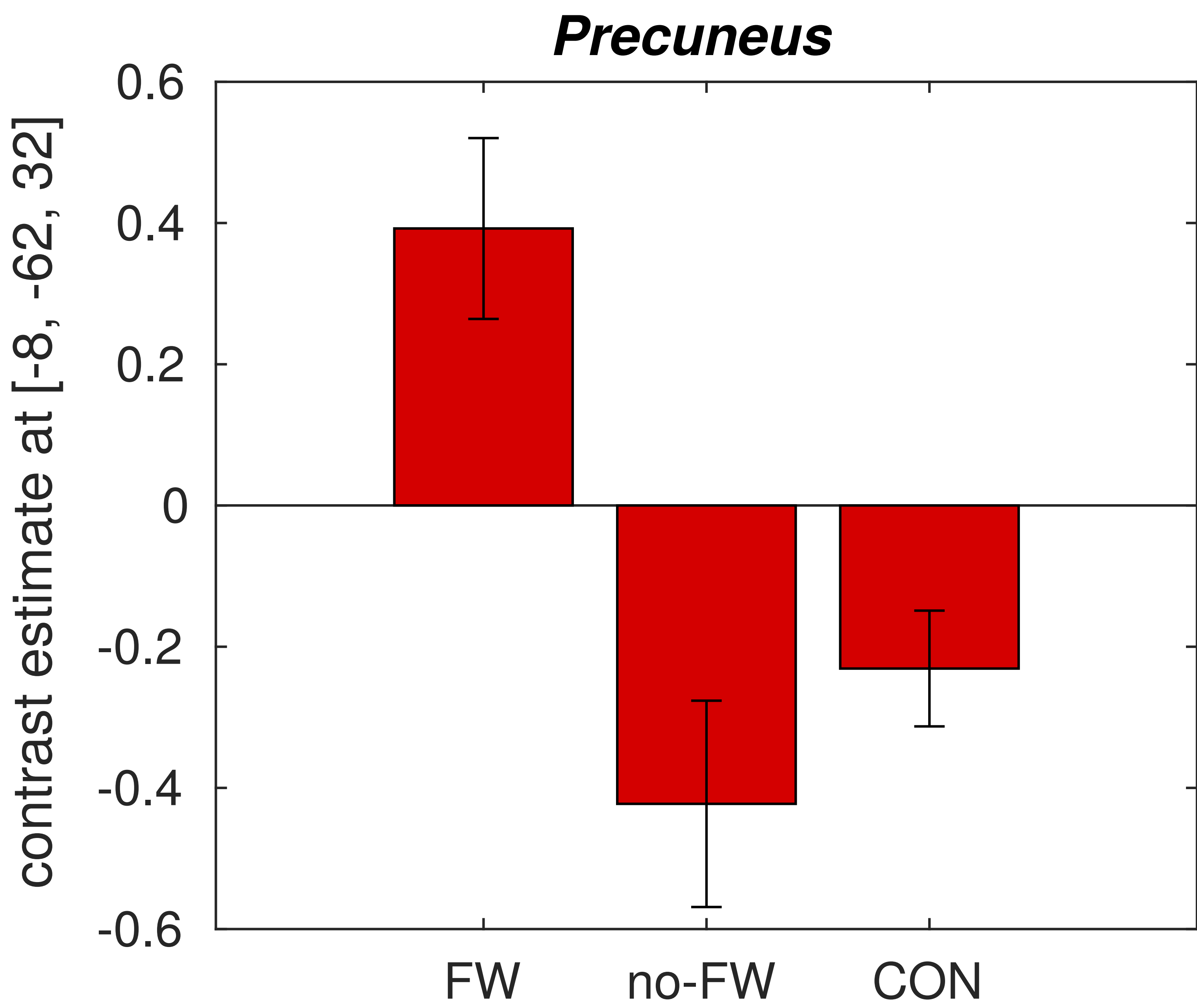

Supplement: Supplementary data 1 [file mmc1.pdf]

no-FW < CON

**A: GCOR**

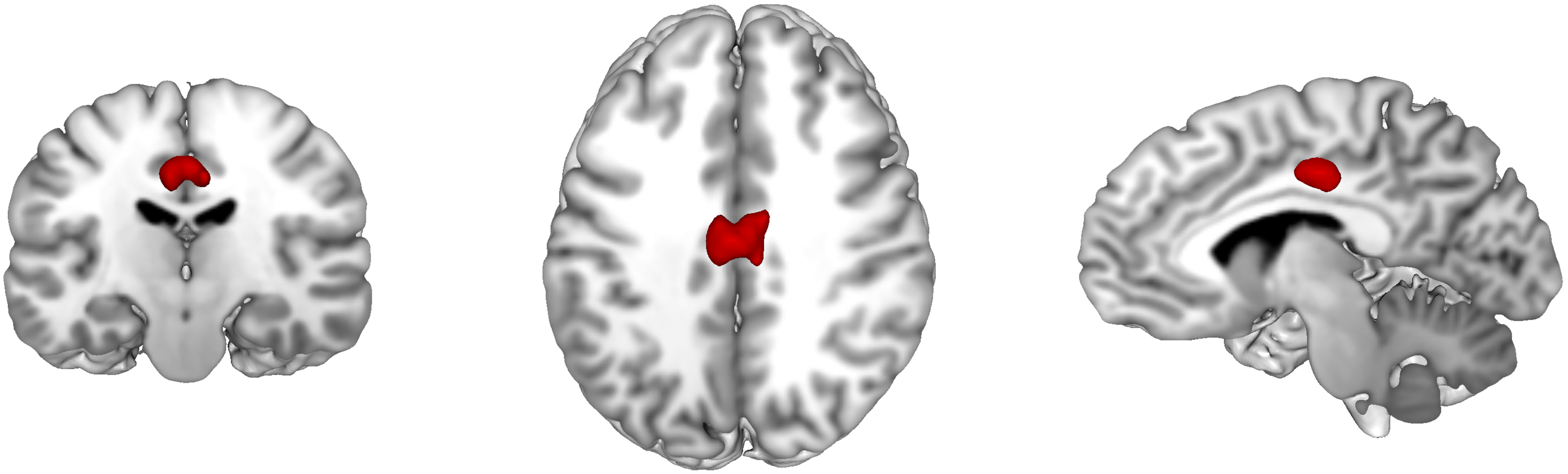

**B: ECM-ADD**

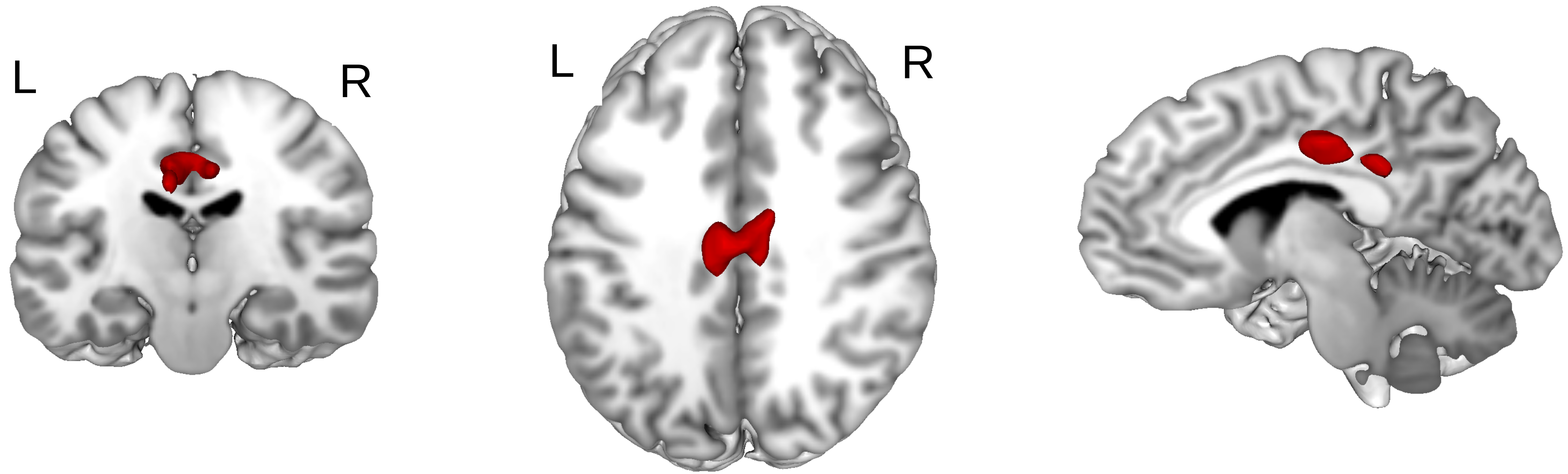

**C: ECM-RLC**

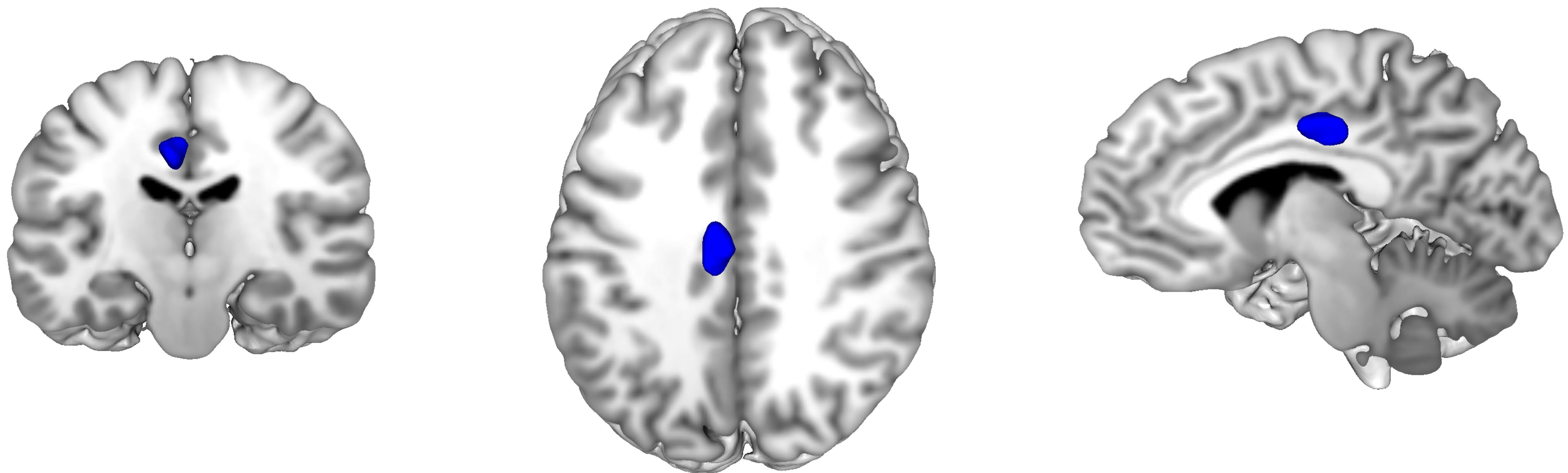

$y = -18$

$z = 36$

$x = -6$

**D: ICC**

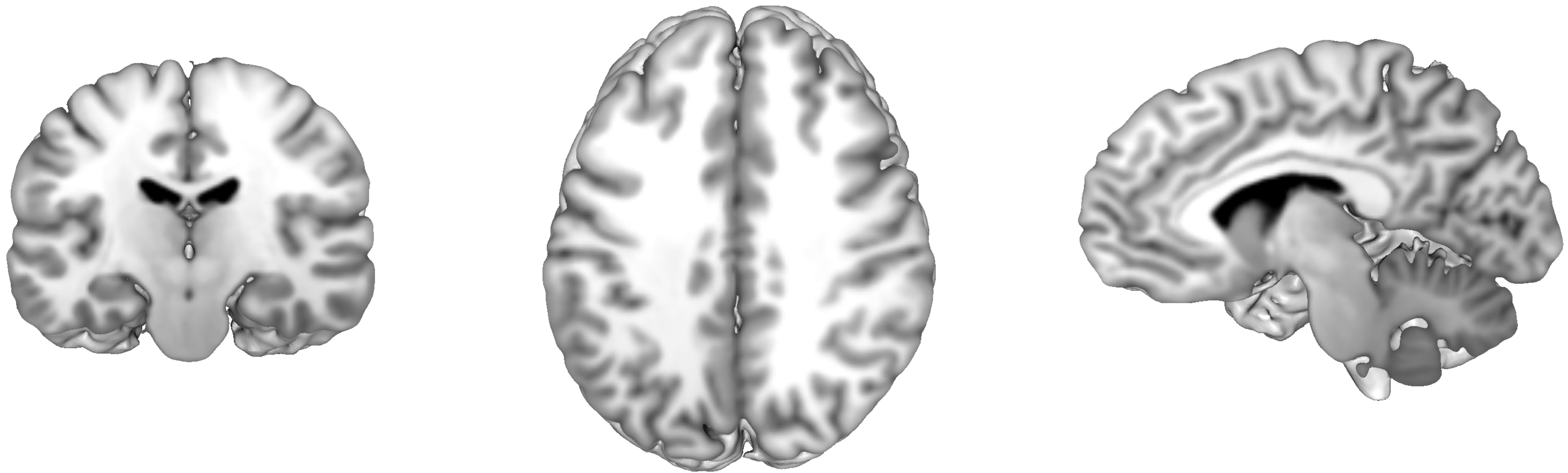

$P < 0.05$  FWE

Supplement: Supplementary data 2 [file mmc2.pdf]

# Seed-based correlation: FW > no-FW+

**A: Left TPJ**

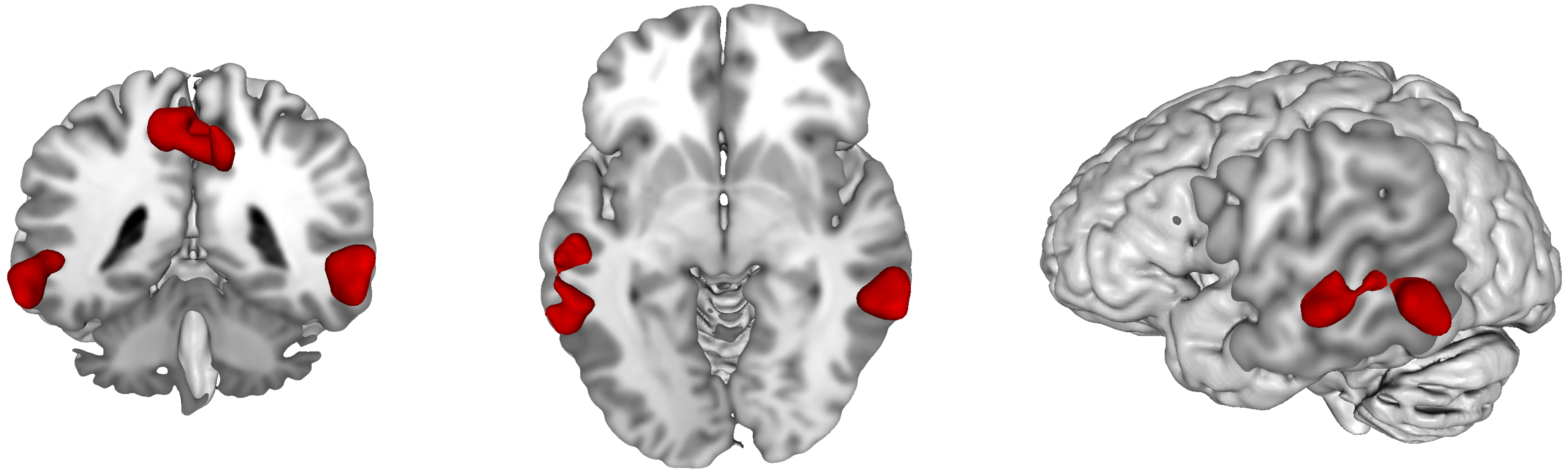

**B: Precuneus**

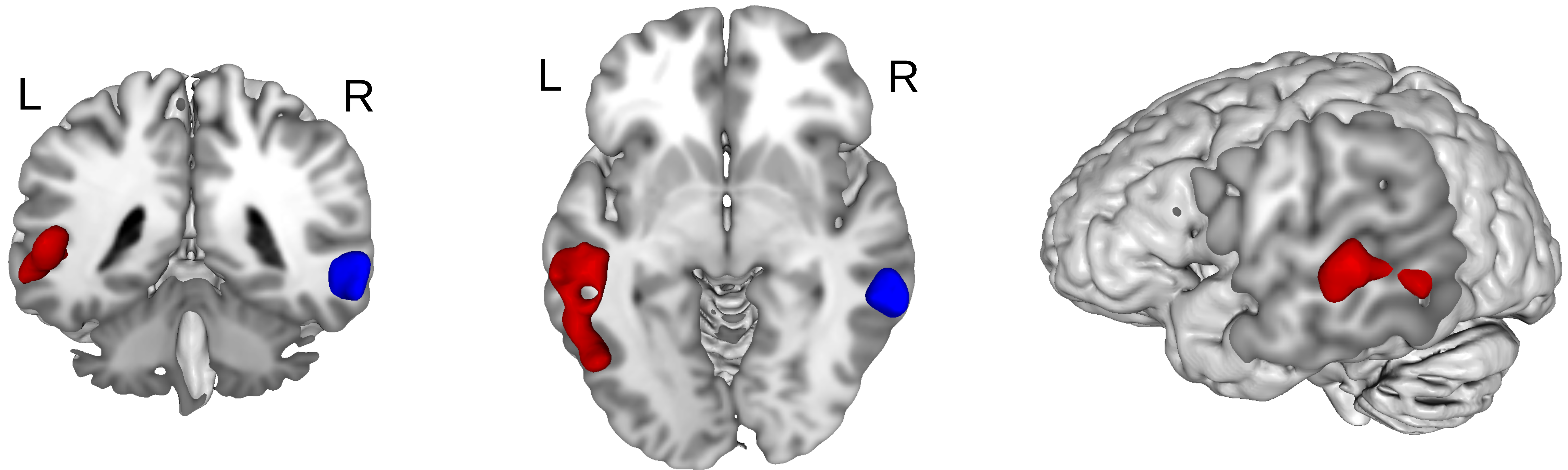

y = -40

z = -2

x = -58

Supplement: Supplementary data 3 [file mmc3.pdf]

corr(GCOR,SFMDRS)

A: FW

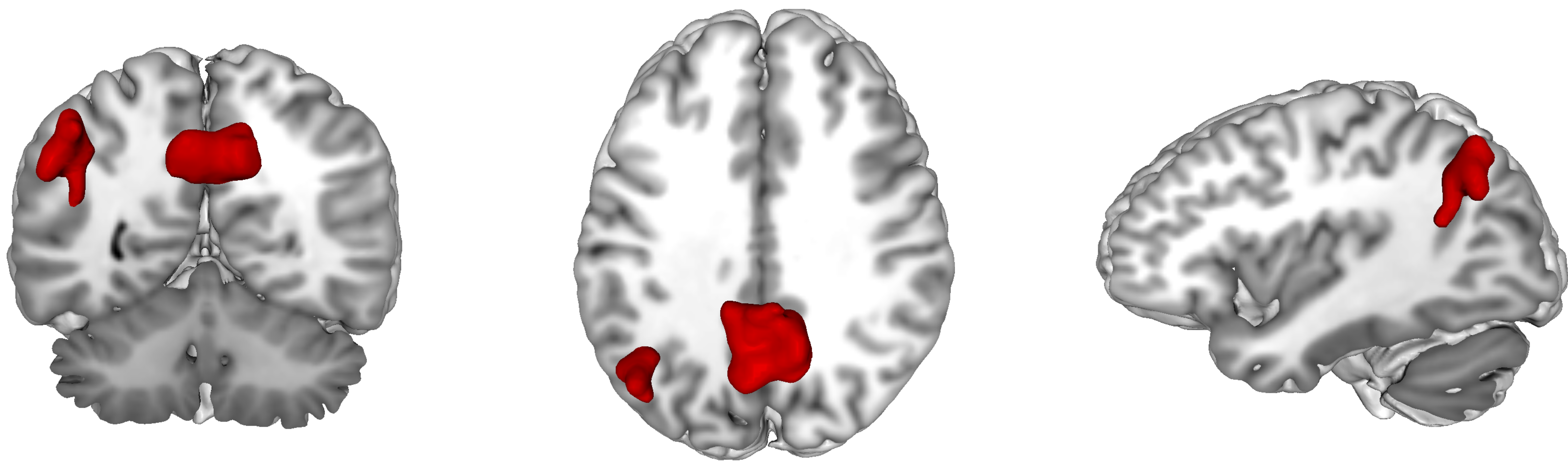

B: no-FW

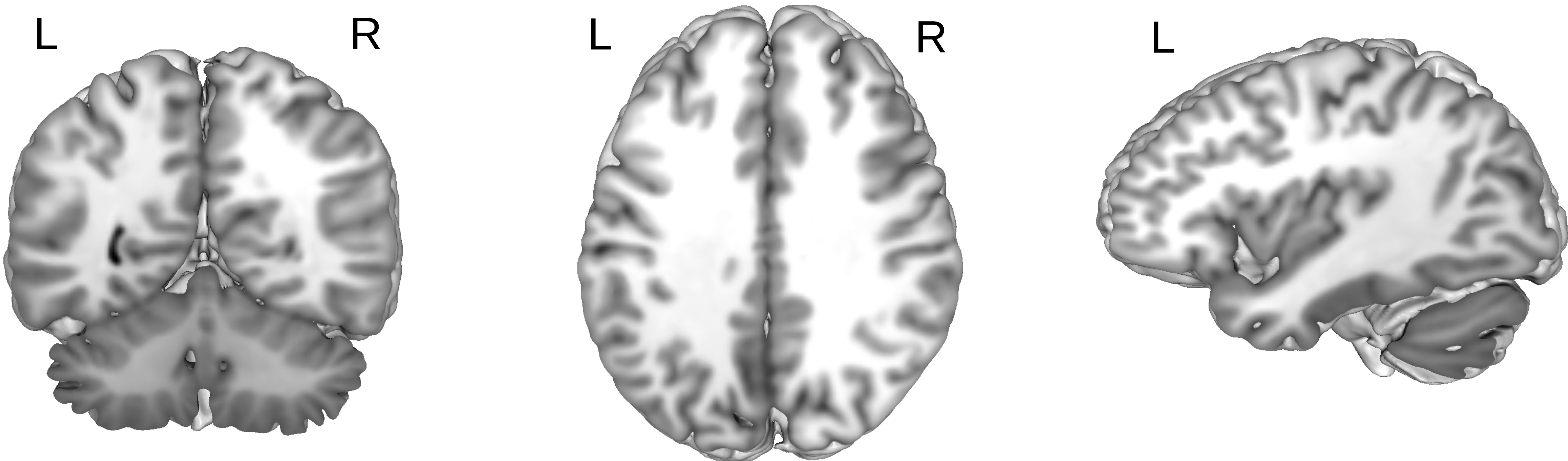

C: FW – no-FW

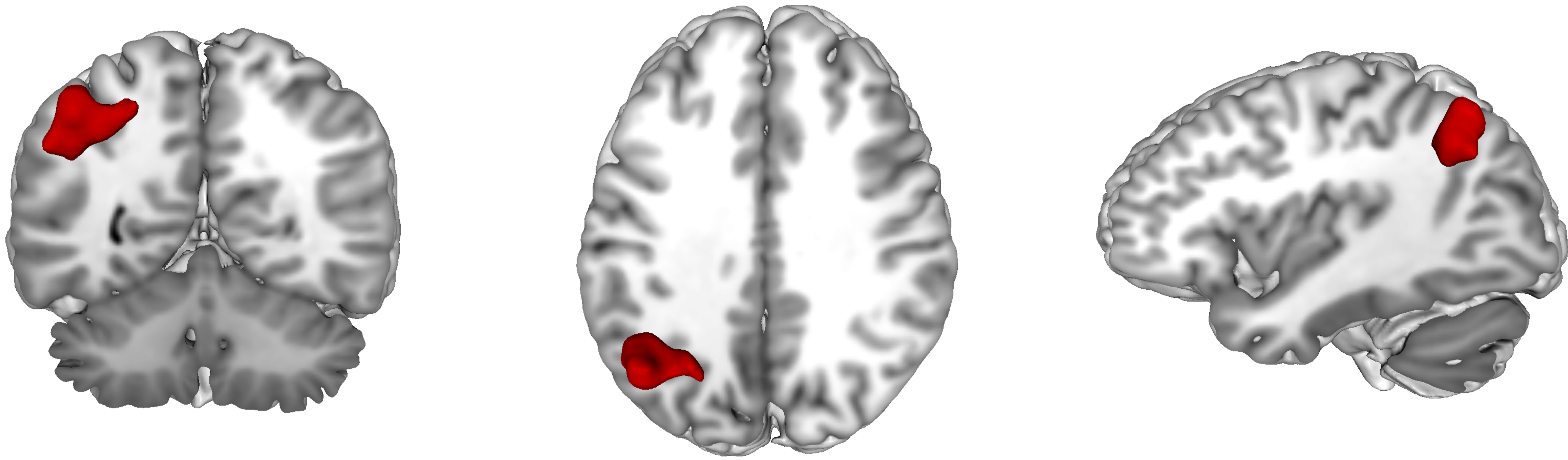

$P < 0.05$  FWE

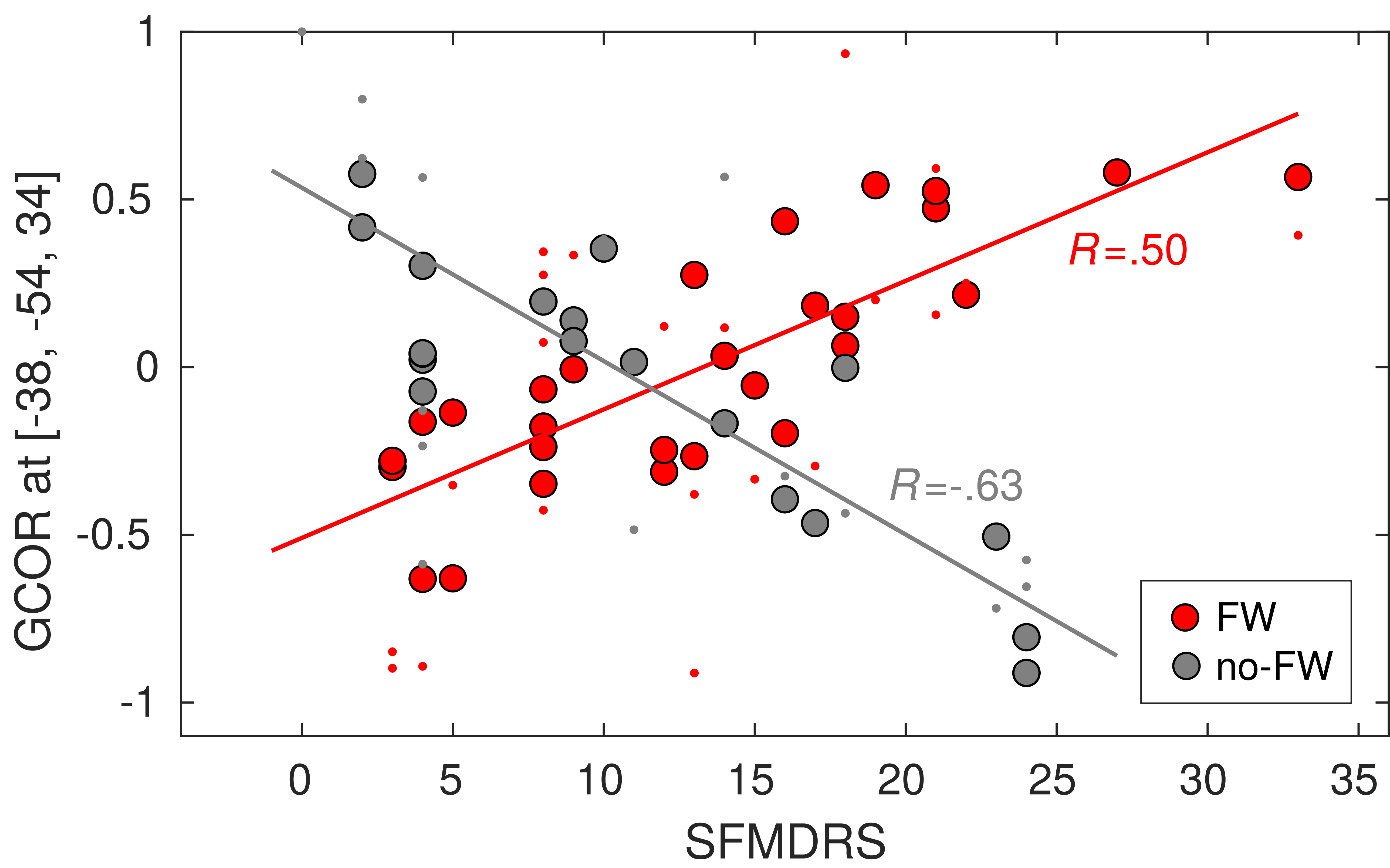

Supplement: Supplementary data 4 [file mmc4.pdf]
