# Supplementary material for: Symptom-severity-related brain connectivity alterations in functional movement disorders
Source: Neuroimage Clin. 2022 Mar 3;34:102981. doi: 10.1016/j.nicl.2022.102981 (PMC8921488; doi:10.1016/j.nicl.2022.102981)
Supplement: Supplementary data 5 [file mmc5.pdf]

Captions for Supplementary Figures

Supplementary Figure S1. Brain network centrality increase with functional weakness (FW) using global correlation (GCOR). The figure shows the same content as Figure 1, but when skipping nuisance regression during image pre-processing. [Significant GCOR differences were found in the precuneus and in the left temporoparietal junction (TPJ) comparing patients with and without FW (**A:** FW>no-FW, red color). Similar GCOR differences were found comparing FW patients and healthy controls in the same anatomical regions but without correction for multiple comparisons (**B:** FW>CON, blue color). The same result was obtained when comparing FW patients with all participants showing no FW (**C:** FW>no-FW+, red color). Bar plots show contrast estimates for the maximum voxel in the left TPJ and the precuneus. Significant results are shown in red with $P<0.05$ using family-wise error (FWE) correction at cluster-level. x, y, z – coordinates in mm; L – left; R – right.]

Supplementary Figure S2. Brain network centrality differences between patients with functional movement disorders showing no functional weakness (no-FW) and healthy controls (CON). Compared with healthy controls, no-FW patients showed a centrality decrease in the posterior cingulate cortex with global correlation (**A**: GCOR) and both measures of eigenvector centrality (**B:** EC-ADD and **C:** EC-RLC, respectively). Results with GCOR and EC-ADD were shown with $P<0.05$ using family-wise error (FWE) correction at cluster-level (red color) while the result with EC-RLC was only obtained without correcting for multiple comparisons (blue color). Note that no result was obtained using intrinsic connectivity (**D**: ICC) even when using an uncorrected threshold. x, y, z – coordinates in mm; L – left; R – right.

Supplementary Figure S3. Orthogonal brain sections showing increased seed-based connectivity with functional weakness (FW) using the left temporoparietal junction (TPJ) and the precuneus as seed-regions. Comparing between participants with FW and without FW (no-FW+) revealed a significant FW-related connectivity increase between both seed regions (**A**: left TPG; **B**: precuneus) and the left and right middle temporal gyrus (MTG). In addition, when using the left TPG as seed region (**A**:), FW-related connectivity increase was also obtained between left TPG and precuneus. Significant results are shown in red with P<0.05 using family-wise error (FWE) correction at cluster-level. Using the precuneus as seed region (**B**:), the right MTL was only obtained without FWE correction (color-coded in blue). x, y, z – coordinates in mm; L – left; R – right.

Supplementary Figure S4. Positive correlation between global correlation (GCOR) and the simplified functional movement disorder rating scale (SFMDRS) in patients with functional weakness (FW). The figure shows the same content as Figure 4, but when skipping nuisance regression during image pre-processing. [(**A:**) Within the group of FW patients, a significant positive correlation between GCOR and SFMDRS was obtained in the precuneus and in the left temporoparietal junction (TPJ). (**B:**) No significant correlation was obtained in the group of patients showing no FW (no-FW). (**C:**) A significant interaction between the factors GROUP (FW/no-FW) and SFMDRS was found in the left TPJ. Significant results are shown in red with P<0.05 using family-wise error (FWE) correction at cluster-level. x, y, z – coordinates in mm; L – left; R – right. The dot-plot on the bottom shows the GCOR-values in the left TPJ for the FW group (in red color) and for the no-FW group (in gray). The bigger dots show the fitted GCOR values within the statistical model while the smaller dots show the zero-mean GCOR values.]
